# British Medicinal Plants

**Published:** 1890-08

**Authors:** Alfred Heath

**Affiliations:** 114 Ebury St., London, England


### BRITISH MEDICINAL PLANTS.

ALFRED HEATH, M. D., F. L. S.

### RANUNCULACEÆ (CONTINUED).

*Helleborus viridis* (Green Hellebore, Bear's Foot, found in thickets or chalky soil; comparatively scarce in England, but abundant where it occurs).—This, though not a showy flower, is, like all hellebores, a very elegant one, bearing yellowish green *spreading* flowers, well worth gathering, not too large, as the well-known Christmas rose (*Helleborus niger*). A short account of this will be found in Dr. Allen's *Materia Medica.* It produces, amongst other symptoms, the following: "Ringing and roaring in the ears, with feeling of stoppage; violent itching in the nose, with frequent and violent sneezing, from application to the parts, pricking on the tongue, relieved by rinsing with water. At first a profuse secretion of saliva and mucus in the mouth, soon followed by a feeling of dryness and of warmth in the pharynx and stomach, which gradually becomes a dull burning. Abdomen sensitive and distended; profuse diarrhœa, with intense colic and tenesmus; great nausea and inclination to vomit; violent headache and thirst; condition bordering on torpor, lasting the whole night, and preventing refreshing sleep; heat at times over the whole body." I found this plant in great abundance some years ago. It extended over a space of about a hundred square yards at the bottom of a wooded hill. I had never seen it growing wild before, and it was a sight worth seeing, especially to a botanist, and the pleasure experienced was intensified at the time by the fact that I was in need of a good quantity of the root.

*Helleborus fœtidus* (Stinking Hellebore, Tetterwort).—Found in similar situations to the foregoing (*Helleborus viridis*). These are the only two members of this genus indigenous to this country. The leaves of the stinking hellebore are said to have extraordinary powers as a remedy for worms. The root has an extremely fœtid smell and bitter taste, and is also very acrid, and if chewed will excoriate the mouth. It is purgative and emetic, and very injurious in large doses. According to the proving in Allen's *Homœopathic Materia Medica*, it produces "violent vomiting and purging, with pain in the stomach, soreness of the mouth and throat; colic, fatal convulsions and swooning; falling off of the hair, and of the nails of the fingers and toes; peeling of the scarf-skin of the whole of the body; restless sleep; profuse discharges from the ulcerated surfaces by applying the drug; difficulty in reading in the evening by candlelight for four days."

*Helleborus niger* (Christmas Rose).—This well-known plant is not indigenous to Britain, but has been so long one of our garden favorites, and moreover is so well known as a homœopathic medicine of the first order—there being a good proving of the drug in Jahr's *Materia Medica* and also in Hering's *Guiding Symptoms*—that I have thought it advisable to mention it with English plants. The ancients esteemed this plant as a powerful *remedy* in *mania.* In more recent times it has been used empirically as a drastic purgative, and in smaller doses as a diuretic and emmenagogue, also in *mania, coma, dropsy,* worms, and psora. The " proving " of this drug on the healthy shows why it is good in certain forms of insanity, etc., etc. It *produces,* among other systems, melancholy, taciturnity; excessive and almost mortal anguish; nostalgia, or a vehement desire to revisit one's country, attended with melancholy, loss of appetite, and sleep. The Swiss are said to be much subject to this affection, as well as other mountaineers. The plant in question is a native of the mountainous parts, especially of Switzerland, Italy, etc. It also produces indolence, sobbing, lamentation, obstinate silence, mistrust, stupidity, weakness of memory, etc. It *produces* involuntary watery diarrhœa, colic, urging, straining, and vomiting; suppression of the menses; drowsiness, constant somnolence, torpid sleep; ulcers and various skin troubles. Suppression of urine; over-distention of the bladder; frequent urging to urinate, with scanty discharge; sudden watery swelling of the skin of the whole body; anasarca. It is one of the most homœopathic remedies in certain forms of dropsy. Allopaths use it because it does good, but knowing nothing of our law of cure, which guides us in the selection of a remedy, they know not why it cures, and therefore can only give it experimentally and often improperly.

*Aquilegia vulgaris* (Columbine).—Found in woods and thickets, etc., chiefly on a calcareous soil. The wild plant is called *Aquilegia* from the shape of the nectaries, which are curved like the talons of an eagle. It should always be employed for homœopathic tinctures. The aquilegia is a very old and favorite garden-plant. This plant was formerly employed as a remedy for eruptions on the skin, for jaundice, scurvy, etc. It is very similar to *Secale cornutum* in its action in child-birth. It is used in Spain as a remedy for stone. Some mention is made of this drug in Dr. Peter's *Diseases of Females.*

114 Ebury St., London, England.

